# Explaining the social gradient in smoking and cessation: the peril and promise of social mobility

**DOI:** 10.1111/1467-9566.13039

**Published:** 2019-11-25

**Authors:** Frances Thirlway

**Affiliations:** ^1^ Department of Sociology Wentworth College University of York York UK; ^2^ Anthropology Department Durham University Durham UK

**Keywords:** smoking cessation, health inequalities, class, social mobility, Bourdieu, gender

## Abstract

Smoking in high‐income countries is now concentrated in poor communities whose relatively high smoking prevalence is explained by greater uptake but above all by lower quit rates. Whilst a number of barriers to smoking cessation have been identified, this is the first paper to situate cessation itself as a classed and cultural practice. Drawing on ethnographic research carried out in a working‐class community in the North of England between 2012 and 2015, I theorise smoking cessation as a symbolic practice in relation to the affective experience of class and social mobility. I show that ambivalence about upward mobility as separation and loss translated into ambivalence about smoking cessation. The reason for this was that the social gradient in smoking operated dynamically at the level of the individual life course, i.e. smoking cessation followed upward mobility. A serious health problem was an appropriate reason to quit but older women continued to smoke despite serious health problems. This was linked to historical gender roles leading to women placing a low priority on their own health as well as the intergenerational reproduction of smoking through close affective links with smoking parents.

## Introduction

Tobacco use in high‐income countries is characterised by a social gradient whereby socioeconomic status is inversely associated with smoking (Barbeau *et al*. 2004a, Blackwell *et al*. 2014, Hiscock *et al*. 2012). Although poor people are also more likely to become smokers, the strongest association is with persistent smoking, i.e. not giving up (Reid *et al*. 2010, US Department of Health & Human Services 2014). Smokers’ principal reasons for quitting are the cost of buying tobacco and the risk to health (Pisinger *et al*. 2011, Vangeli and West 2008) and barriers to cessation amongst lower socioeconomic status (SES) smokers include reduced social support for quitting, normative smoking in poor communities, low motivation to quit, stronger addiction and targeting by the tobacco industry (Barbeau *et al*. 2004b, Hiscock *et al*. 2012, Twyman *et al*. 2014). The importance of cultural factors in relation to smoking has been recognised (Goldade *et al*. 2012, Nichter 2003, Nichter *et al*. 2009, Poland *et al*. 2006), but few studies have considered cessation itself as a classed or cultural practice. There is a tendency in public health to equate ‘culture’ with risk factors, i.e. forms of ‘irrational’ behaviour by the cultural Other (Bradby and Nazroo 2010, DiGiacomo 1999), whereas dominant health practices such as smoking abstention or cessation are not seen as culturally specific but are identified as the ‘rational’ choice. According to this perspective, it is continued smoking, not cessation, which demands explanation. In this paper I will theorise smoking cessation as a symbolic practice in relation to the affective experience of class and social mobility. I will use the term working‐class throughout to indicate people engaged in manual and routine jobs, but I recognise that these are only classes ‘on paper’ (Bourdieu 1985) and that class is essentially relational (Bourdieu 1990: 50, Bourdieu and Wacquant 1992: 15).

In his famous account of contrasting classed dispositions, French theorist Bourdieu described a working‐class ethic of sociable hedonism which he saw as going hand in hand with the rejection of middle‐class practices seen as ‘pretentious’, one example of which was paying excessive attention to one's health or appearance (Bourdieu 1984: 180–4). He argued that non‐participation in working‐class hedonism – which I suggest might include ceasing to smoke – was read as a betrayal because *difference could only arise from the desire to distinguish oneself, that is, from refusal or repudiation of the group* (Bourdieu 1984: 381). Despite his own rural working‐class origins, Bourdieu paid limited attention to the moral and emotional implications of class (Sayer 1999) although late in his career he described his ambivalence about being elected to the prestigious College de France and his feeling that his success was a form of treachery towards his father (Bourdieu 1990: 129). It has largely been left to feminist theorists to describe how alongside sociable hedonism and pride in working‐class culture (Skeggs 2004) runs a parallel strand of shame and trauma of becoming aware of oneself as ‘lacking’ through being made to feel inappropriate (Fox 1994: 13). This dictates a desire to escape, but also creates ambivalence because wanting something more than what your parents had implies that there is something wrong with their life and therefore also with them (Walkerdine *et al*. 2001: 158, Steedman 1987, Lawler 2000, Reay 2017: 114). The stigma of poverty leads to a tension between wanting to reject respectability and to be respectable, between aspiration and rejection of dominant values (Sayer 2002). The ‘injuries of class’ (Sennett and Cobb 1972) produce mixed emotions: resentment at the undeserved and valuable advantages conferred by an accident of birth, suspicion that some dominant values and behaviours have no intrinsic worth beyond signposting middle‐class status, and temptation to refuse to acknowledge any value to the goods monopolised by the dominant class, leading to self‐exclusion from potential advantages (Sayer 2002); this is described by Willis in relation to working‐class boys resisting their schooling (Willis 1977). My argument in this article is that working‐class ambivalence about the benefits of upward mobility produces a similar ambivalence towards smoking cessation as a practice closely associated with it.

## Methods

My approach was naturalistic enquiry and specifically ethnography, which studies cultural practices in particular places over a relatively long time period (Messac *et al*. 2013: 184) and seeks to *understand another life world using the self – as much of it as possible – as the instrument of knowing* (Ortner 2006: 42). My research took place between 2012 and 2015 in a small village in a rural, formerly coal‐mining area of the North East of England. A high percentage of people worked in routine and manual occupations, educational qualification levels were low and there were high levels of ill health and worklessness (NOMIS 2018). Increasing spatial segregation by income (Fahmy *et al*. 2011, Quillian 2012) means that the social gradient in smoking maps to spatial inequalities (Glenn *et al*. 2017, Pearce *et al*. 2012, Thompson *et al*. 2007): a third of research participants smoked, in line with prevalence rates for manual and routine workers at the time, which were and remain much higher than in higher SES groups (West *et al*. 2019). The sample was a convenience one and included 67 women and 52 men; the age range was 18–100 with a mean age of 62 for the whole sample and 58 for current smokers; ages given in the text are ages in 2012. All research participants were white UK nationals; most migration since the early 20th‐century has been out of the area to seek better economic opportunities elsewhere. Ethical approval for the research was obtained from the Durham University School of Health Ethics Committee and the Anthropology Department Ethics Committee. I talked research participants through an information sheet which explained the purpose of my study before obtaining written consent (ASA 2011). Research locations have been obscured and names and other details of participants changed in the text to protect anonymity.

Most of my 150 visits to the village were made in 2012 and 2013 when I visited the village two to three times a week, usually staying for 3–5 hours. I generally went to the Working Men's Club on Friday afternoons and for old‐time dancing once or twice a week, I attended elderly lunch‐club sessions once or twice a week and the church, chapel and hairdressing salon intermittently. I also volunteered at the primary school and attended one‐off events such as summer fairs, fundraising events, band displays and funerals and talked to 26 individuals or couples in their homes. Data were collected in the form of audio recordings and field notes and quotes in this paper are taken from these. Smoking data formed only a small proportion of the total; broader life histories, everyday events in the village and its history going back to the 1930s were recurring topics. Ethnographic analysis proceeds not so much by thematic analysis as by taxonomies and classifications, looking at what people have in common and how they differ (Prentice 2010). I compared individual histories of smoking and quitting in the context of broader life stories, local history and value systems, or what Kleinman calls local moral worlds (Kleinman 2010: 375). I treated individual histories analytically as units rather than fragmenting them into thematic categories (Mishler 1996, Riessman 2008: 12) before generalising to theoretical perspectives (Melia 2010).

## Findings

### Smoking cessation and upward mobility: a problematic link

I pointed out to Benjamin (23) the incongruous sight of a tiny woman in her seventies craning her neck to speak to a six‐foot drag queen at the bar, but he was distracted – he was waiting to go for a cigarette with Shelley (23), who had been on her phone for 15 minutes. She told me later that night: *Benjamin's a bad smoker, he'll go out with you for a cigarette, and 5 minutes later he'll go out with someone else for another one*, and he agreed ruefully. A packed Working Men's Club was enjoying a fundraising show but as the evening went on, it became clear that one of the three drag queens on stage was much less charismatic than the other two. As a result, tables of smokers who had been taking randomly staggered smoking breaks now waited until she started a solo number, at which point half the hundred‐strong audience got up and went outside for a cigarette.

Of the 47 I knew at this event, 23 were smokers including 12 of the 14 people on Benjamin and Shelley's table: occasions like this made apparent not only how many people smoked, but how much smoking was tied in with sociability. Former factory operative Hazel (66) had given up smoking for 10 months several years before we spoke*: I was on night shifts, I just got sick of it, the machines were very slow, you could smoke everywhere, you made yourself sick … everybody on the night shift smoked, except one person*. She had started smoking again on New Year's Eve; she told me: *Someone I worked with said ‘oh, just have one’*. Kevin (59), who propped up the club bar every Friday afternoon, went back to smoking after 12 years because of pressure from fellow miners, although he did manage to quit again later on. Ex‐soldier Nicholas (57) smoked a pipe when I visited him for an interview but told me he had previously stopped for 4 years, until his smoking mother ‘forgot’ he had given up and bought him a new pipe and tobacco for Christmas. Even if ex‐smokers stayed quit, they showed little sign of the distaste for their former habit whereby tobacco becomes disgusting or abject, making relapse less likely (Bell 2011, Thompson *et al*. 2009). Former cook and barmaid Rita quit smoking in her forties after a heart attack, but she still felt at risk of relapse in her seventies, as we shall see in a later section.

While these examples illustrate normative smoking, ethnography involves interpretative depth (Panter‐Brick and Eggerman 2018) rather than a paraphrase of what people say (Fassin 2013); it can take many years to understand an ethnographic event (Bourdieu and Wacquant 1992: 164). Following close engagement with wider issues of class and stigma in the village, I gradually realised that continuing to smoke involved not just an affective attachment to working‐class sociality, but also rejection of middle‐class pretention and ambivalence towards upward mobility (Skeggs 2005: 975, Skeggs 2004: 88, Skeggs 2011: 506). This was not something that could be discussed explicitly; class is notoriously difficult to talk about because the arbitrariness and injustice of its effects lead to defensiveness or resentment (Sayer 2002). Sometimes hints emerged: retired pitman Peter (85) was teased at the Club for *talking posh* to impress me, and shop worker Shelley (23) told me that the *popular kids* at school had smoked at her school whereas *the swots* did not. Peter's efforts to transcend local ways of speaking and ‘the swots’ failure to smoke were a problem because they involved a distancing from their own community. The reason peer pressure was applied was that upward mobility separates people; I will argue that smoking cessation can have the same effect.

Builder Malcolm (47) did not envy residents of the middle‐class neighbourhood down the road, who had not enjoyed themselves *the way us lot have*; his parents and siblings all worked in manual jobs, except for one older brother who *worked hard at his books* and had gone into an office job and moved away from the village; this brother was also the only one of the family who did not smoke. His mother Hazel (66) later told me that he gave up in his thirties – but *he never smoked properly anyway*, she told me, laughing and mimicking smoking without inhaling. Whilst she was proud of her elder son's achievements, her alienation from his upward mobility was apparent from her mocking his failure to smoke ‘properly’ – unlike, she implied, those who still belonged to the village such as herself and her other children. Smoking cessation was potentially pretentious because it was demonstrably associated with upward social mobility which itself led to geographical mobility, both of which involved either a literal or a symbolic distancing from class and community of origin. There were both practical reasons for those who could afford it to move out of the village, i.e. limited housing stock and public transport, and symbolic reasons in that it had a poor reputation as a rough or lawless place. This meant that people born into working‐class families who later moved into middle‐class occupations not only gave up smoking but also moved out of the village: smoking status operated as a symbol of social differentiation and was linked to physical separation (Salazar and Smart 2011). Two of the children of care worker Frances (65) and her husband, a former miner, were employed as a postal worker and shop worker, lived in the village and smoked, whereas their third child had trained as a social worker, moved away from the village and did not smoke. A couple in their sixties never told people which village they were from when they were growing up because it was *a bit dodgy*; later they *moved up in the world* and stopped smoking. Although they eventually moved back to the village, their social discomfort was evident from their fear of being burgled; the outside of the house they owned bristled with CCTV warnings, in contrast to other older residents whose door was always unlocked and who invited me to let myself in.

When I visited miner's widow Ivy (88) at her home one evening, she prepared a cup of tea and an elaborate plate of biscuits for me before recounting her life story, including how she came to stop smoking at the age of 49 whilst staying with her sister Pamela's daughter:

The way it happened was we used to stay with my niece for Christmas, I went to bed one night and my husband said I'll just have a smoke, when he came up I said have you put it out … I was so worried, with it not being my house, that I stopped smoking at that minute, I don't know what it was.

Ivy also told me how as a child, she had failed the examination to attend the academic grammar school rather than the secondary modern whereas her sister Pamela had passed. Pamela went into a skilled occupation, moved away and stopped smoking, and neither of her children subsequently smoked. In the light of the contrasting social trajectories of the two sisters, Ivy's decision to stop smoking during a visit to her niece hinted at complex feelings about class, family and her own ability to ‘live up’ to her relatives. School selection was still a keenly‐felt aspect of social differentiation in the village; failing to *pass for the grammar* was a painful memory for those who reached the age of eleven before 1979, when the local selective system was replaced by comprehensive schools. Peter liked to remind me that he had passed the examination although he did not attend the grammar school in the town *because all my friends were in* [the village] *– I wanted to go with my mates*.

Ambivalence towards upward mobility was reproduced in the smoking and cessation practices of younger generations: office worker Rebecca (26) had moved away to get her young daughter into a better school; asked about the village, she told me *I do like it, it's home, but I don't want to live there, I was pleased to move out – though if anybody criticises it I get very defensive of it*. She initially told me she didn't smoke, then looked embarrassed and said *well actually I am a bit of a social smoker, I occasionally have one – but I'm not really a smoker*. Rebecca demonstrated her ambivalence in relation to her class position by following the practices of middle‐class ‘secret’ smoking (Choi *et al*. 2010, Glenn *et al*. 2017). Her partner Adam (30), torn between his hard‐drinking Club friends and his aspirational young family, took a different route by purchasing an electronic cigarette, which allowed him to demonstrate sociable hedonism in the Club whilst adopting more middle‐class norms in relation to health (Thirlway 2018).

### Older women continuing to smoke: a family affair

Whilst ambivalence about upward social mobility translated into ambivalence towards smoking cessation, a serious health problem was always an appropriate reason to quit and did not expose the quitter to critique. Research participants stopped smoking in response to an acute illness such as a heart attack or stroke, after a cancer diagnosis, after developing a chronic condition such as chronic obstructive pulmonary disorder (COPD) or diabetes or in some cases following a health scare or the illness of a close relative. I was struck, however, by the number of older women who continued to smoke despite serious health problems. When I spotted Phyllis at the Club one night after several months absence, she told me she had only recently come out of hospital because of her emphysema, but she continued to smoke: *I'm 74, I've been smoking 62 years – not much point giving up now*, she said. Nicholas (57) described how his late father had stopped smoking because of his wife's angina whereas she – now in her eighties – was still smoking when I interviewed him. Nigel (54) smoked from the age of 9 until 49 when he was diagnosed with diabetes and stopped immediately. His wife continued to smoke even after two operations for bowel cancer: *She won't smoke outside, so my clothes stink*, he complained.

Men were far more likely than women to describe the process of smoking cessation as a sudden, almost violent break. Some of the expressions they used were: *I just stopped … threw the pack in the fire … threw down the pack … never had another one*. This may be because men were less willing to admit that they had struggled to quit, preferring to describe their cessation as a triumph of the will (Thirlway 2016, White and Baird 2013), but several women also thought quitting was easier for men; Kim told me that *men can just stop* and Yvonne (69) was impressed with her husband's self‐control when they both gave up many years before:

Every day I was over the moon because I hadn't been to the shop and bought any, but him, when he gave up he had a packet in his top pocket for weeks, once he took the wrapper off, one time he undid the top, but he never had one.

Gail (52) managed to stop smoking but she had to use a nicotine inhaler for more than a year to cope with the cravings. Her husband had been pressing her to give up because of a bad cough and she was in fact later diagnosed with COPD. He contrasted his own, earlier and apparently instant quit with hers, and criticised her use of an inhaler. *She's still smoking them things*, he complained.

I have previously suggested that one reason why older women with serious health problems continued to smoke was what Warin *et al*. have described as a relational sense of identity which placed their own health at the bottom of a hierarchy of concerns (Warin *et al*. 2008: 104, Thirlway 2016). Retired home carer June (65) was typical, spending all her time caring for siblings and grandchildren, shopping for her father or driving him to family events and appointments, but continuing to smoke despite COPD. June's devotion to her family reproduced her own mother's role at a time when miners’ families were large, the pits depended on women's labour to feed, wash and launder for the men, and the effects of food insecurity on children during short‐time working, strikes and lockouts were probably buffered by mothers reducing their own food intake (McIntyre *et al*. 2003, Piperata *et al*. 2013). June's smoking was itself a link to the past (Robinson and Holdsworth 2013: 59), to her mother Iris who had died of lung cancer at the age of 52, and to her grandmother Daphne, also a heavy smoker. When June's father Ronald (87) came home on leave at the end of the Second World War, Daphne heard in the Club that he had cigarettes and went round to his house with her daughter Iris as they were all *choking for a smoke*. Ronald liked to tell this story, and always concluded by saying: *I got my wife for fifty Senior Service* [cigarettes]. It was often adults who were or had been particularly close to their smoking parents who failed to give up even after a diagnosis of serious or chronic illness. Cleaner Kim (46) had a number of health problems and had tried everything to quit. She did not smoke when she was with her partner, but as soon as she saw her mother *or even if I think about her* she craved a cigarette. Returning to my point about the problematic association of smoking cessation with upward mobility, Kim was also part of the first generation to attend the former selective grammar school after it was amalgamated with several secondary moderns to form a comprehensive school; she had painful memories of the *snooty* teachers looking down on the first non‐selective pupils from the mining villages, and of how the older, selective pupils distinguished between *them and us*.

The context of the gendered reproduction of maternal smoking was that couples tended to settle in the female partner's home area so that daughters remained geographically and emotionally close to mothers, not least for practical help with childcare (Clapson 1998, Young and Willmott 1957). As well as working part‐time into their seventies, many older women I met cared for grandchildren in their homes for several days a week; this was the only way that parents could work, as paid childcare was unaffordable on their wages (Fergusson *et al*. 2008, Gray 2005, Ward *et al*. 2007). Hazel's young granddaughter came round to her house at a quarter to seven every morning when her mother caught the bus to work. Her 15‐year‐old grandson had lived with her since he was eight, a traditional arrangement in the area (Chaplin 1971).

Some daughters who were close to their smoking mothers *had* managed to quit, but found it a huge struggle and were constantly at risk of relapse. Rita (75) was one of these, as she told me at a village hall event:

My father smoked a pipe, my mother smoked Embassy Regal … she didn't stop even when she had hardened arteries. I found it hard to quit – I did it 21 years ago after I had a heart attack, but when I was in hospital I told my daughter to take my cigarettes away, as I knew that if I had them, I would go out for a cigarette. I haven't had a one since – it's like alcoholics, if I had one I would be back on them. If I had 6 months to live, I would go back to them.

An emotional link to parents through smoking was less apparent amongst men who continued to smoke, which may be a function of typical gender roles; the few men I met who remained unmarried and were close to their (smoking) parents, taking on the role of kinkeepers carrying out the emotional labour involved in maintaining family ties (Brown and Derycke 2010) did also seem to find it particularly hard to quit.

Whilst the parent–child relationship appeared to be key in most cases, occasionally it was a connection to people other than parents which gave smoking a strong emotional pull: Nicholas (57) was taught to smoke a pipe by his grandfather; Jeanette (60) worked in a male environment where *all the men hated me*, until her eventual acceptance by her colleagues was signalled by their offering her cigarettes. Benjamin (23) also struggled to stop smoking; his parents were non‐smokers but he was close to his heavy‐smoking grandmother, with whom he had lived for several years.

## Conclusions

Whilst qualitative research involves findings about relatively small numbers of people in specific places, when properly combined with theoretical analysis it can generate insights which significantly advance the field. I started with the observation that smoking was normative in many contexts of my fieldwork; it was linked to sociability and peer pressure played a key role in smoking relapse. The importance of social norms in smoking is well‐known but does not explain how the social gradient developed, i.e. why smoking norms changed in some groups but not others. I have described in previous work how cigarettes were taken up initially by the middle‐class, then abandoned as part of a new concern with health and fitness (Ferrence 1989, 1996, Pampel 2005, Pampel *et al*. 2010, Poland 2000: 10). As the middle‐class moved away from smoking, a circular process took place whereby smoking became ever more stigmatised in middle‐class circles, leading to ever more middle‐class cessation (Thirlway 2018). In this paper I asked why the working‐class continued to smoke; I found that ambivalence about upward mobility as separation and loss translated into ambivalence about smoking cessation because the social gradient operated dynamically at the level of the individual life course, i.e. smoking cessation followed upward mobility.

This was the ambivalence described by Rebecca, who wanted to ‘get away’ from the village but was quick to defend it from those who criticised it – an ambivalence which has been described as divided or ‘cleft’ habitus (Bourdieu 2007: 100, Friedman 2016) or hybridity (Bhabha 2012 [1994], Lucey *et al*. 2003), and which translated into Rebecca's secret smoking, pulled between different classed perspectives on smoking and quitting. For those who struggled to quit and for those who relapsed, smoking cessation was a battle which was never won because it was closely tied to emotive issues of class and family allegiance and betrayal; sometimes symbolic meanings make health behaviours which appear self‐evidently positive into something much more complex and potentially threatening (McKie *et al*. 2003, Stead *et al*. 2011, Warin *et al*. 2008). I am not characterising the maintenance of unhealthy behaviours as a form of *resistance* to middle‐class norms (Eakin *et al*. 1996, Factor *et al*. 2011, Poland and Holmes 2015) or a reversal of values consequent on being found wanting by the middle‐class (Skeggs 1997, Willis 1977). Defining working‐class scorn for pretention as resistance rather than ambivalence risks doing one of two things. It can either represent resistance as irrational, missing the fact that although middle‐class status and the practices associated with it are desirable in terms of material affluence and security, they are problematic in their cultural otherness and removal from friends, family and neighbourhood (Ortner 2006: 31, Lawler 1999). Alternatively, it can celebrate resistance for its own sake, missing the stigma of poverty and the desire to escape and potentially lapsing into a complacent excusal of inequality or an idealisation of working‐class culture (Sayer 2005: 120, McRobbie 2002).

My analysis suggests that tobacco control messages which associate smoking cessation with middle‐class aspiration in general and a preoccupation with one's own future health in particular may contribute to working‐class ambivalence about smoking cessation. What then is the alternative? We know that low‐SES smokers are more likely than high‐SES to cite current health problems rather than worries about future health as triggers to quit, and also more likely to cite the expense of smoking (Pisinger *et al*. 2011, Vangeli and West 2008). I have suggested elsewhere that the cost of smoking, especially as it relates to the diversion of financial resources from the family to a habit negatively constructed as addiction, is an important moral consideration for working‐class smokers and explains the turn to consumption of the cheapest possible tobacco as involving more than mere affordability (Thirlway 2019a). Raising the minimum price of tobacco products (Gilmore *et al*. 2010, Hiscock *et al*. 2012, Thirlway 2019b) and framing cessation messages around financial cost may be a way forward (Pisinger *et al*. 2011). As I have argued elsewhere, there may also be a role for switching to e‐cigarettes as a way for smokers to maintain their involvement in working‐class sociality and hedonism whilst reducing the risk to their health (Thirlway 2016, 2018).

My second finding was that although actual illness – as opposed to an excessive and potentially pretentious preoccupation with health – was a legitimate reason to stop smoking, older women with chronic health conditions failed to quit. We know that many seriously ill people continue to smoke (Jonsdottir and Jonsdottir 2007, Wilson *et al*. 2011) but there is some question as to whether women generally find it harder to quit smoking than men (Amos *et al*. 2012, Hiscock *et al*. 2012). Recent studies have found that men over the age of 45 or 50 were more likely to quit than women (Jarvis *et al*. 2013, NHS National Services Scotland 2018) and my research suggested two possible reasons for this. The first was historical gender roles leading to women placing a low priority on their own health and the second was close links to smoking parents, particularly mothers: smoking cessation appeared to be particularly hard both for women who replicated their mothers’ role as family caregiver, and for those with close emotional ties to (smoking) mothers or other close relatives, even long after those relatives had died. We know that children are more likely to smoke if parents do (Gilman *et al*. 2009, Kandel and Wu 1995, Leonardi‐Bee *et al*. 2011, Loureiro *et al*. 2010, Melchior *et al*. 2010), and particularly if mothers do (McAloney *et al*. 2014). There is also some evidence that where one parent smokes but not the other, children follow gendered models (Gilman *et al*. 2009), with daughters particularly likely to follow smoking mothers. My argument is that the correlation relates to closeness of relationship and proximity in space as well as continuity of role as family caregiver. This is particularly relevant to working‐class women since middle‐class smokers are less likely to have memories of parental smoking because the social gradient in smoking is now of long standing (Feldman and Bayer 2011), and the geographical mobility which disrupts the intergenerational reproduction of smoking is largely an elite practice (Holdsworth 2009). Intergenerational ties to smoking mothers provide a further illustration of my argument that smoking cessation involves the risk of emotional loss and separation from family and class. The importance of family factors (Johnston and Thomas 2008, Varcoe *et al*. 2010) and the continuing influence of older generations on younger smokers have been underestimated and a new emphasis on increasing rates of smoking cessation amongst older women and promoting multi‐generational cessation needs to replace the long‐standing policy focus on young people.

This study has a number of limitations. Whilst I did speak to younger people, most of the people I saw were older because they were more likely to be present in most of the community settings I had access to and generally had more time to talk to me. This may explain why I came across few examples of younger people stopping smoking at key transitions over the life course such as forming a stable partnership, obtaining stable employment or starting a family (Bricard *et al*. 2017, McDermott *et al*. 2004, 2009, Measham *et al*. 2011). It was also the case that younger people who had stopped smoking in such circumstances (such as the adult children of older research participants) had moved away, and in that sense my social network in the village, if not the village itself constituted a ‘smoking island’ (Christakis and Fowler 2008, Thompson *et al*. 2007). It may be, however, that rates of smoking cessation in younger age groups in working‐class communities are in fact very low; documented quit attempts are highest in 45–54 year olds, and older age groups are most likely to succeed (NHS England 2019). Although there is appreciable cessation amongst people in their twenties, this is much less the case among people with fewer years of education and those with smoking parents (Chassin *et al*. 1996). In relation to the wider applicability of my study, it might also be suggested that former mining communities represent a very specific type of working‐class community, such that the ambivalence about social mobility and the patterns of intergenerational reproduction of smoking which I have described are not applicable elsewhere; however, I would argue from recent research in urban areas of North West England that at least in those neighbourhoods which have not been regenerated or gentrified, working‐class family and community networks such as those I have described continue to operate (Thirlway 2019a, Ward *et al*. 2007), to shield their members from the worst effects of austerity and neoliberalism.
